# *Streptococcus pneumoniae* capsule determines disease severity in experimental pneumococcal meningitis

**DOI:** 10.1098/rsob.150269

**Published:** 2016-03-23

**Authors:** Lucy J. Hathaway, Denis Grandgirard, Luca G. Valente, Martin G. Täuber, Stephen L. Leib

**Affiliations:** Institute for Infectious Diseases, Faculty of Medicine, University of Bern, Bern 3001, Switzerland

**Keywords:** *Streptococcus pneumoniae*, meningitis, serotype, capsule

## Abstract

*Streptococcus pneumoniae* bacteria can be characterized into over 90 serotypes according to the composition of their polysaccharide capsules. Some serotypes are common in nasopharyngeal carriage whereas others are associated with invasive disease, but when carriage serotypes do invade disease is often particularly severe. It is unknown whether disease severity is due directly to the capsule type or to other virulence factors. Here, we used a clinical pneumococcal isolate and its capsule-switch mutants to determine the effect of capsule, in isolation from the genetic background, on severity of meningitis in an infant rat model. We found that possession of a capsule was essential for causing meningitis. Serotype 6B caused significantly more mortality than 7F and this correlated with increased capsule thickness in the cerebrospinal fluid (CSF), a stronger inflammatory cytokine response in the CSF and ultimately more cortical brain damage. We conclude that capsule type has a direct effect on meningitis severity. This is an important consideration in the current era of vaccination targeting a subset of capsule types that causes serotype replacement.

## 1. Background

*Streptococcus pneumoniae* is one of the most important human pathogens, responsible for 14.5 million episodes of serious disease, including meningitis, pneumonia and sepsis, per year and 826 000 deaths in children under 5 years of age [1]. It is the most frequent cause of bacterial meningitis in children and is particularly associated with severe disease with a high mortality rate and brain damage leading to neurological sequelae in up to 50% of survivors [2–5]. The bacteria may be surrounded by one of more than ninety known types of polysaccharide capsule that determine the serotype and some of which are targets for the vaccines currently available [6]. While some serotypes are common, usually asymptomatic, colonizers of the human nasopharynx, others are associated with invasive disease [7–9]. However, when serotypes usually associated with colonization do invade, the disease they cause tends to be particularly severe [10,11]. Here, we address the question of whether capsule type affects severity of disease directly.

*In vivo* models of experimental meningitis have shown that strains vary in their ability to cause disease, but this has often been attributed to the cell wall rather than the capsule [12,13]. In such studies where the strains differ in serotype and genetic background, it is difficult to conclude which factor(s) is responsible for differences in the severity of resulting disease. Another group has proposed that the capsule serotype, rather than the genetic background, is the most important factor for determining the course and severity of pneumococcal meningitis [14]. In order to study the effect of capsule in isolation from the genetic background, we produced mutants in which the capsule operon had been deleted or replaced by that of another strain [15]. Switching serotype has been shown previously to change accessibility of protein ligands leading to differences in binding to epithelial cells and virulence in the respiratory tract [16] and virulence in a murine intraperitoneal infection model and a chinchilla otitis media model [17,18], but the effect on meningitis is unknown. Our aim in this study was to determine whether changing the capsule type of a pneumococcal strain alone would change the disease severity in an infant rat meningitis model as determined by the ability of the bacteria to multiply and induce an inflammatory cerebrospinal fluid (CSF) response, brain damage and mortality.

## 2. Material and methods

### 2.1. Bacterial strains

A clinical nasopharyngeal isolate of *S. pneumoniae* (106.66, serotype 6B) was collected during a nationwide surveillance programme [8,19]. The capsule operon was removed and replaced with a Janus cassette [20,21] creating non-encapsulated mutant 106.66Janus. The Janus cassette was then replaced by its own capsule operon to make backtransformant 106.66cps106.66 (serotype 6B) or the capsule operon of another nasopharyngeal clinical isolate, 208.41, to produce mutant 106.66cps208.41 (serotype 7F), or clinical isolate B109.15 (isolated from the blood of a patient with pneumonia) to produce mutant 106.66cpsB109.15 (serotype 7F) as described previously [15]. Visualization of capsule thickness was performed by fluorescence isothiocyanate (FITC)-dextran exclusion assay as described previously [15] where the zone of exclusion of FITC-dextran (2000 kDa, Sigma) indicates the polysaccharide thickness.

### 2.2. Infant rat model of pneumococcal meningitis

A well-established infant rat model of bacterial meningitis [22] was modified as follows. The bacteria were used in the animal model without prior passage *in vivo.* Litters of 12 nursing Wistar rats with their dams were obtained from Charles River (Sulzfeld, Germany) and acclimatized for 5 days. Eleven-day-old rats weighing 27.1 ± 8.3 g were infected intracisternally with 10 µl 0.85% NaCl containing 2.5 × 10^6^ colony forming units (CFU) ml^−1^ live *S. pneumoniae*. Animals were randomly assigned to receive bacterial strain 106.66 (*n* = 36), 106.66Janus (*n* = 6), 106.66cps106.66 (*n* = 6), 106.66cps208.41 (*n* = 30) or 106.66cpsB109.15 (*n* = 6). Bacterial meningitis was confirmed by quantitative analysis of bacterial cultures in samples of CSF at 21 hours post-infection (hpi), when the rats developed symptomatic disease. Antibiotic therapy with ceftriaxone (Rocephine^®^, Roche Pharma, Basel, Switzerland; 100 mg kg^−1^ d^−1^, i.p.) was administered at 21 and 29 hpi.

To assess severity of disease, the rats were weighed and examined clinically at 0, 21, 29 and 45 hpi and scored as described previously [22]. Animals not able to stand upright were immediately sacrificed by an overdose of pentobarbital (Esconarkon, Streuli, Uznach, Switzerland; 150 mg kg^−1^, i.p.) for ethical reasons. Spontaneous mortality was also documented.

Punctures of the cisterna magna were performed to obtain CSF using a 30-gauge needle at 21 and 45 hpi. CSF samples not used for bacterial titres or to visualize capsule thickness were centrifuged at 16 000*g* at 4°C for 10 min and the supernatants frozen at −80°C for later determination of cytokine concentrations. Animals were sacrificed with an overdose of pentobarbital (150 mg kg^−1^) at 45 hpi and perfused with 4% paraformaldehyde (PFA) in PBS before the brains were removed and fixed in PFA for histological analysis.

### 2.3. Cytokine measurement in cerebrospinal fluid

The concentrations of the cytokines IL-6, IL-1β, TNFα, IL-10 and IFNγ in the CSF samples were determined using the microsphere-based multiplex assays (MILLIPLEX^®^ MAP kit, rat cytokine/chemokine magnetic bead panel, Millipore Corporation, Billerica, MA) as described previously [23].

### 2.4. Histomorphometrical analysis of brain damage

Brain damage was quantified as previously described in animals sacrificed at 45 hpi [22,23]. Volume of cortical damage (areas of decreased neuronal density or cortical necrosis) was expressed as a percentage of the total cortical volume investigated in at least 16 brain sections per animal. Apoptosis in the hippocampus was quantified by counting cells showing features of apoptosis (condensed, fragmented dark nuclei, apoptotic bodies) in four slices spanning the hippocampus of both hemispheres. Cells were counted in three fields of view in each of the two blades of the dentate gyrus and a mean value per animal was calculated.

### 2.5. Preparation of bacteria and measurement of growth

Bacteria were prepared for inoculation by growing to mid-log phase in brain heart infusion broth (BHI; Becton Dickinson and Company, le Pont de Claix, France). To quantify bacterial growth, CFU were quantified. Serial dilutions of CSF samples from infected rats were made in 0.85% NaCl, plated out onto CSBA plates and incubated overnight at 37°C with 5% CO_2_. CFU were counted the next day.

### 2.6. Statistical analysis

Statistical analyses were performed using GraphPad Prism software (Prism v. 6 for Windows, GraphPad Software Inc., San Diego, CA). The D'Agostino & Pearson omnibus normality test was used to discriminate between data following a normal Gaussian distribution or not. An unpaired Student's *t*-test was used for normally distributed data; otherwise, Mann–Whitney test was applied. Spearman correlations were calculated to obtain *r*-values. Mortality rates were calculated using log-rank (Mantel–Cox) test for significance based on successfully infected rats and numbers of animals sacrificed due to ethical reasons or dying spontaneously. A value of *p* < 0.05, two-tailed, was considered statistically significant.

## 3. Results

### 3.1. Serotype 6B capsule was associated with greater mortality due to meningitis than serotype 7F

Infant rats were inoculated intracisternally with either a wild-type clinical pneumococcal isolate, 106.66 (serotype 6B; *n* = 36), its non-encapsulated mutant (106.66Janus; *n* = 6) or mutant 106.66cps208.41 (*n* = 30) in which the 6B capsule has been replaced by a capsule of serotype 7F. The bacterial strains were not passaged in animals prior to infection. Both the serotype 6B and 7F strains were able to establish an infection in all rats, with the exception of one rat that received the 7F strain. However, no bacteria could be recovered from the CSF of rats that received the non-encapsulated mutant 106.66Janus and they exhibited no signs of disease. Mean bacterial titre recovered from CSF at 21 hpi was 7.1 × 10^8^ CFU ml^−1^ for strain 106.66 (6B) and 1.7 × 10^8^ CFU ml^−1^ for strain 106.66cps208.41 (7F). This was not significantly different (*p* = 0.23). However, the value for 106.66 may be an underestimate as it does not include values for the rats that died earlier than 21 hpi, which may have had higher titres. The same pattern of slightly higher titre of 6B than 7F in CSF at 21 hpi was also seen for the experiment shown in the electronic supplementary material, figure S1, with a mean of 7.9 × 10^8^ CFU ml^−1^ for the backtransformant 106.66cps106.66 (6B) and 3.3 × 10^7^ CFU ml^−1^ for a different serotype 7F capsule switch mutant 106.66cpsB109.15 (7F).

**Figure 1.**
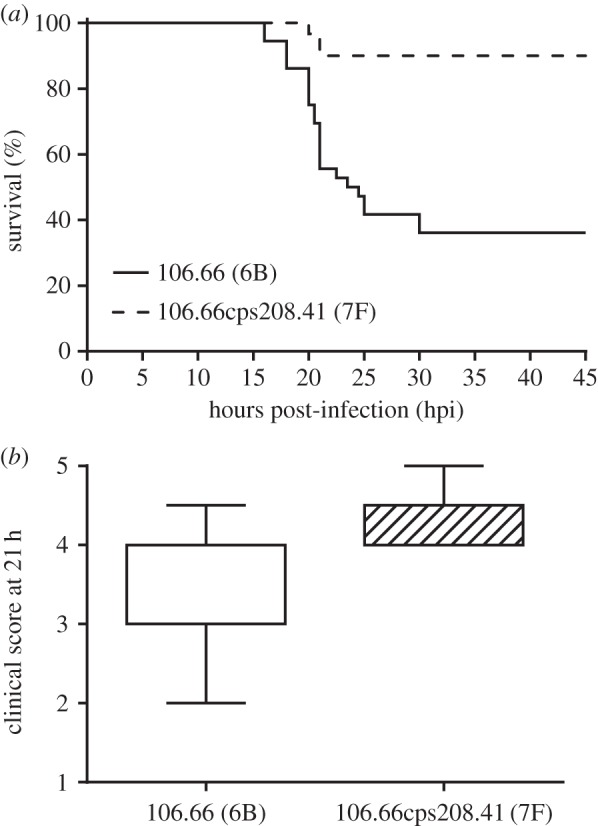
Clinical parameters assessed during acute bacterial meningitis in animals infected by intracisternal inoculation with pneumococci. Switching the capsule of wild-type strain 106.66 from serotype 6B to 7F (creating capsule switch mutant 106.66cps208.41) caused (*a*) a significant increase in survival rates over 45 h (*p* < 0.0001) and (*b*) a significant reduction in severe disease (higher clinical score) at 21 h (*p* < 0.0001).

Survival of the rats over 45 hpi was significantly greater for those which had received the 7F serotype strain than those which received the strain of serotype 6B (*p* < 0.0001; figure 1*a*). At 21 hpi, the time of administration of antibiotic, the mean clinical score, indicating health, was also greater for the serotype 7F than the serotype 6B strain (*p* < 0.0001; figure 1*b*). There was no difference in weight change between the groups receiving the serotype 6B strain and the 7F strain at 21 hpi (97.1% and 97.4% of initial weight respectively) or 45 hpi (95.8% and 96.7% of initial weight respectively). Greater survival of rats receiving serotype 7F than 6B was also seen in an additional experiment when strain 106.66cps106.66 (serotype 6B) or strain 106.66cpsB109.15 (serotype 7F) were administered (electronic supplementary material, figure S1).

### 3.2. Serotype 6B caused more cortical injury than serotype 7F

**Figure 2.**
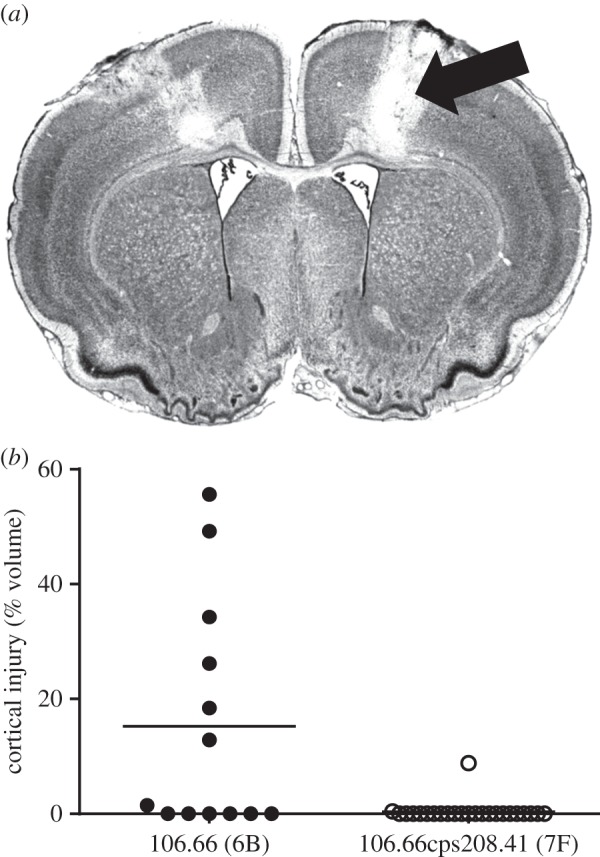
Cortical injury was quantified in rats that survived to 45 hpi. (*a*) Histology section of the brain of a rat exposed to strain 106.66 (serotype 6B), arrow indicates region of cortical damage. (*b*) The volume of cortical injury was significantly less in rats infected with the capsule switch mutant 106.66cps208.41 (serotype 7F) than with its serotype 6B parent strain, 106.66 (*p* = 0.0006).

Of the rats which survived to 45 hpi, 7/13 which received the serotype 6B strain developed cortical damage compared with 2/23 which received serotype 7F (Fisher's exact test, *p* = 0.0049). The extent of cortical damage was also greater in the serotype 6B than the 7F group (figure 2, mean 15.2% versus 0.4% respectively, *p* = 0.0006). This may be an underestimate since rats that died before 45 hpi were not included and these are likely to have the most cortical damage. The number of apoptotic cells in the hippocampus of animals surviving until 45 hpi was also determined, but no significant difference was observed between the two groups (mean values of 2.2 versus 1.0 for the groups which received serotype 6B and 7F, respectively, *p* = 0.246). More cortical damage was also seen with the backtransformant (serotype 6B) than the alternative serotype 7F capsule switch mutant 106.66cpsB109.15 (electronic supplementary material, figure S2). The mean number of apoptotic cells in the hippocampus of rats that survived to 45 hpi was higher in those which had received the backtransformant (serotype 6B) than the serotype 7F capsule switch mutant 106.66cpsB109.15 (5.2 versus 0.09, respectively, *p* = 0.0476 by Mann–Whitney).

### 3.3. Cerebrospinal fluid cytokine levels differed between rats infected with serotypes 6B and 7F

**Figure 3.**
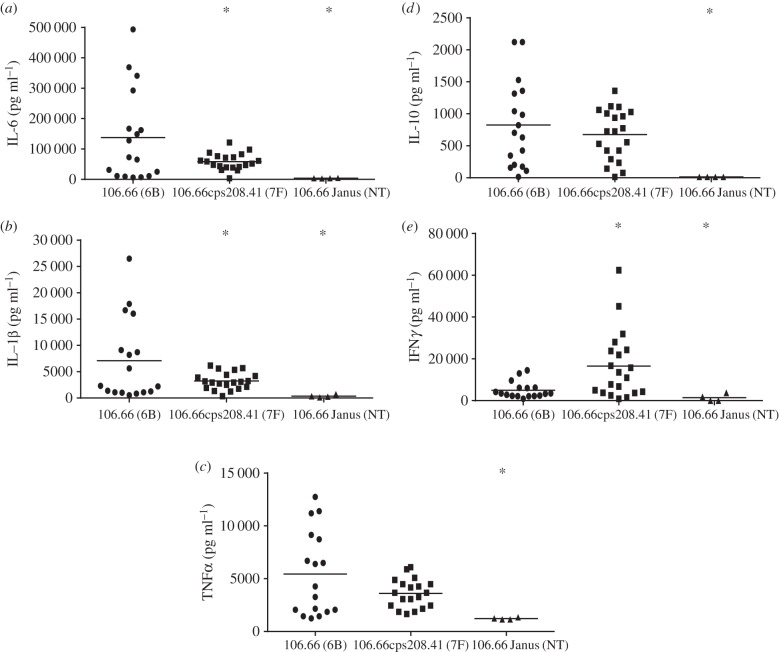
(*a*–*e*) Cytokine concentrations in CSF at 21 hpi. Asterisk indicates significant difference from strain 106.66 (*p* < 0.005).

CSF samples were taken at 21 and 45 hpi and tested for cytokine concentrations. At 21 h, higher concentrations of cytokines IL-6 and IL-1β were found in the CSF of the rats which had received serotype 6B than either the serotype 7F or the non-encapsulated mutant groups (*p* < 0.05; figure 3*a,b*). TNF*α* and IL-10 concentrations were slightly higher following infection with 6B rather than 7F but not significantly, although significantly higher than the non-encapsulated mutant (figure 3*c,d*). In contrast, IFN*γ* concentration was greater in the CSF of the rats that received the serotype 7F strains than either the 6B or the non-encapsulated groups (*p* < 0.05; figure 3*e*). At 21 h, the mean concentration of IL-6 and IL-1β was also higher for the backtransformant (serotype 6B) than the alternative capsule switch mutant of serotype 7F (electronic supplementary material, figure S3), although owing to the low number of animals the data did not reach significance. At 45 hpi, levels of all cytokines tested were below the limit of detection in CSF samples of all rats, regardless of the serotype of the infecting pneumococcal strain (data not shown).

### 3.4. Correlations between bacterial load, cytokine concentrations and cortical damage

**Figure 4.**
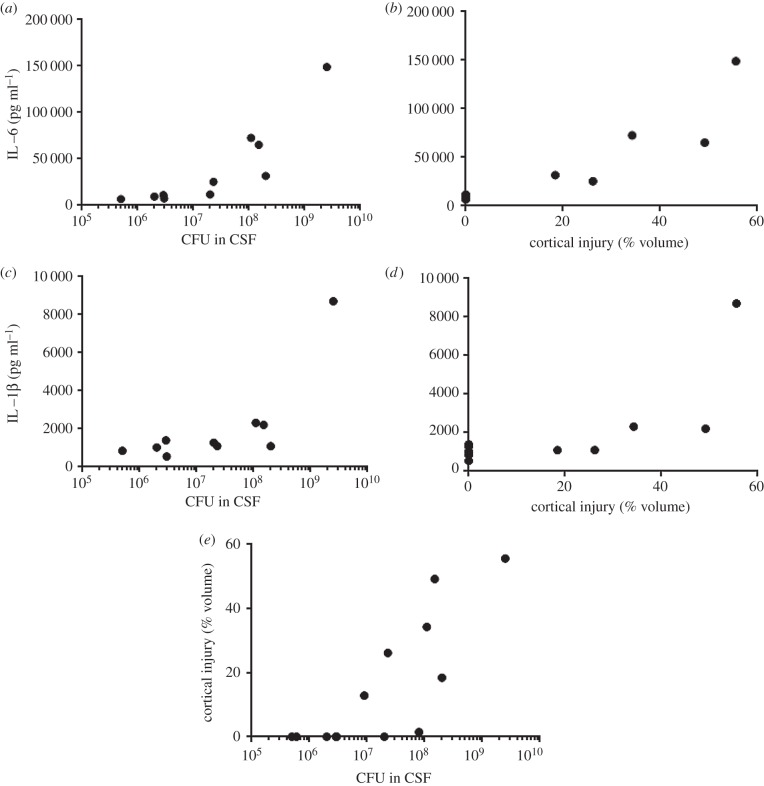
Correlations between bacterial load, cytokine concentrations and cortical damage. (*a,b*) IL-6 concentration in CSF at 21 hpi correlated with (*a*) CFU in the CSF at 21 hpi (Spearman correlation *r* = 0.9152, *p* = 0.0005) and (*b*) percentage of cortical injury at 45 hpi (*r* = 0.9116, *p* = 0.0005). (*c,d*) IL-1β also correlated with (*a*) CFU (*r* = 0.6485, *p* = 0.049) and (*d*) cortical injury (*r* = 0.7435, *p* = 0.0196). (*e*) CFU at 21 hpi correlated with cortical injury at 45 hpi (*r* = 0.8669, *p* = 0.0003).

It was noted that the cytokine concentrations induced by the serotype 6B strain 106.66 in figure 3 were spread over a wide range and so we looked for correlations between bacterial load, cytokine concentrations and cortical damage (figure 4). IL-6 concentration in the CSF at 21 h did correlate with CFU in the CSF (figure 4*a*) and also percentage of cortical injury at 45 hpi (figure 4*b*). The same pattern was seen with IL-1β which also correlated with both CFU (figure 4*c*) and cortical injury (figure 4*d*). This also meant that there was a correlation between CFU and cortical injury (figure 4*e*). Spearman correlation *r*-values ranged between 0.6485 and 0.9152 and all *p*-values were < 0.05.

### 3.5. Serotype 6B, but not 7F, maintained a thick capsule in the cerebrospinal fluid

**Figure 5.**
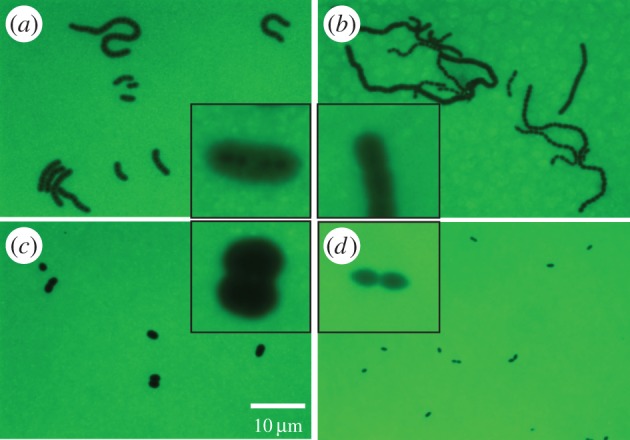
FITC-dextran analysis of capsule thickness. Capsule thickness was determined in wild-type strain 106.66 (serotype 6B) (*a*,*c*) and capsule switch mutant 106.66cps208.41 (serotype 7F) (*b,d*) growing in BHI before inoculation of rats (*a,b*) and in CSF recovered from rats 21 h after infection (*c*,*d*), showing that the serotype 6B, but not the 7F, strain can maintain a thick capsule in CSF. Original magnification = 630×, insets show close ups of outer images. All outer images are to the same scale as each other, and all insets are to the same scale as each other. Scale bar indicates 10 µm and refers to the outer images.

Bacteria from the inoculum, grown in BHI, were viewed microscopically in the presence of FITC-dextran, according to the established method [15], to visualize capsule thickness. Although quantitative measurements were not determined, it was observed that both strains 106.66 (6B) and 106.66cps208.41 (7F) formed chains of relatively thickly encapsulated bacteria during this *in vitro* culture in rich medium (figure 5*a,b*), although some variation in capsule thickness was apparent in strain 106.66cps208.41 (figure 5*b*). However, for bacteria recovered from the CSF at 21 hpi, there was a striking difference in capsule thickness, with strain 106.66 (serotype 6B) able to maintain a thick capsule whereas strain 106.66cps208.41 (7F) had a much thinner capsule (figure 5*c,d*). Both strains in CSF were found predominantly in pairs or individual bacteria rather than in chains. The backtransformant 106.66cps106.66 (6B) also had a thick capsule in both BHI and CSF (electronic supplementary material, figure S4*a,c*), whereas the 106.66cpsB109.15 (7F) had a thinner capsule in both BHI and CSF (electronic supplementary material, figure S4*b,d*). 106.66cps106.66 (6B) and 106.66cpsB109.15 (7F) were also found in pairs or individual bacteria in CSF compared with chains in BHI.

## 4. Discussion

It has been observed that some pneumococcal serotypes are associated with worse clinical outcomes than others [10], but it is unknown whether this is due directly to the capsule type or to other factors of the disease-causing strains. Here, we show that severity of meningitis in an infant rat meningitis model does depend on the capsule type and that a capsule is necessary to establish meningitis.

Serotype 6B strains (wild-type clinical isolate or its backtransformant capsule mutant) caused more severe disease than either of two mutants expressing 7F capsule in the same genetic background. Serotype 6B caused more mortality than serotype 7F and this correlated with more cortical injury, although not significantly more apoptotic cells in the hippocampus. Levels of inflammatory cytokines IL-6 and IL-1β in the CSF at 21 h after infection were higher in rats infected with serotype 6B than 7F, suggesting an association between an exaggerated inflammatory response and worse outcome. This was supported by the finding that within the group of rats infected with the serotype 6B strain, there was a positive correlation between IL-6 and IL-1β concentrations and amount of cortical damage.

TNFα, IL-10 and IFNγ were increased by both serotypes with no difference between them for TNFα and IL-10 and a higher level of IFNγ in the serotype 7F group. Therefore, both capsule types induced expression of all the cytokines tested, but the pattern of response of the different cytokines varied by serotype.

While for most of the cytokines investigated our results are in line with previous findings showing that high levels of cytokines, particularly IL-6, in the CSF, are associated with clinical severity and worsening of neurological long-term sequelae [24,25], we observed a higher level of IFNγ in animals infected with the less virulent 7F serotype. This is in contradiction to recent experimental results describing IFNγ as an important contributor to the pathogenesis of bacterial meningitis [26,27]. One study showed an association between IFNγ-driven acute brain pathology and the long-term neurological sequelae resulting from pneumococcal meningitis using IFNγ knockout mice infected by a serotype 3 strain (WU2). In the other study, the survival of mice infected with a serotype 4 strain (TIGR4) was prolonged upon treatment with a neutralizing antibody directed against IFNγ. It is therefore conceivable that the relevance of IFNγ production in the pathogenesis of meningitis may be serotype-specific or host-specific.

Although there was a difference in disease severity between the two serotypes, both were able to establish an infection in the rat model. This was in contrast to a non-encapsulated mutant that did not establish an infection or cause any detectable disease, indicating that a capsule is essential to establish an infection in this model of meningitis. This is compatible with the fact that pneumococci lacking capsule are rarely associated with invasive disease [28].

Although the reason for the difference between the serotypes in severity of meningitis they cause is unknown, it is of note that there was a striking difference in capsule thickness between the two serotypes when isolated from the infected rats. While serotype 6B maintained a thick capsule in the CSF, 7F had a much thinner capsule. This could be because synthesis of the 6B capsule requires less metabolic effort than the 7F, and this is reflected in capsule thickness under nutrient-restricted conditions as we have shown before *in vitro* [15]. Both serotypes changed from growth in chains in the inoculum to pairs or individual bacteria in the CSF. A thick capsule and reduction in chain length are both mechanisms to reduce complement binding [29].

A strength of this study is that we compare serotypes in the same genetic background, but a limitation is that we have only looked at two serotypes: 6B commonly associated with colonization and 7F with invasive disease, both serotypes included in the Prevnar 13 and Pneumovax 23 vaccines. It would be of great interest to expand the study to other serotypes to see whether serotypes associated with colonization cause more severe meningitis in general than serotypes associated with invasion. Varvio *et al.* [30] described two lineages of serotypes based on evolution of capsule regulatory genes: one ancestral and associated with carriage (including serotype 6B) and the other with laterally transferred sequences associated with invasive disease (including serotype 7F). Although beyond the scope of this study, a comparison of members of the two lineages in the infant rat model may yield interesting findings in the future.

Using mutants with the same genetic background but different serotypes allowed us to determine conclusively that the capsule type affects severity of meningitis. In the current era of vaccination against a subset of capsules, some serotypes are increasing in colonization prevalence as competition from vaccine serotypes is removed [31]. Disease potential of replacement serotypes should be a consideration in vaccine design, because there is the possibility that disease caused by replacement serotypes could be more severe than that caused by serotypes included in the vaccines.

## Supplementary Material

Supplementary Figures

## Data accessibility

The datasets supporting this article have been uploaded as electronic supplementary material.
