# Supplementary material for: Streptococcus pneumoniae capsule determines disease severity in experimental pneumococcal meningitis
Source: Open Biol. 2016 Mar 23;6(3):150269. doi: 10.1098/rsob.150269 (PMC4821241; doi:10.1098/rsob.150269)
Supplement: Supplementary Figures [file rsob150269supp1.pdf]

Supplementary material

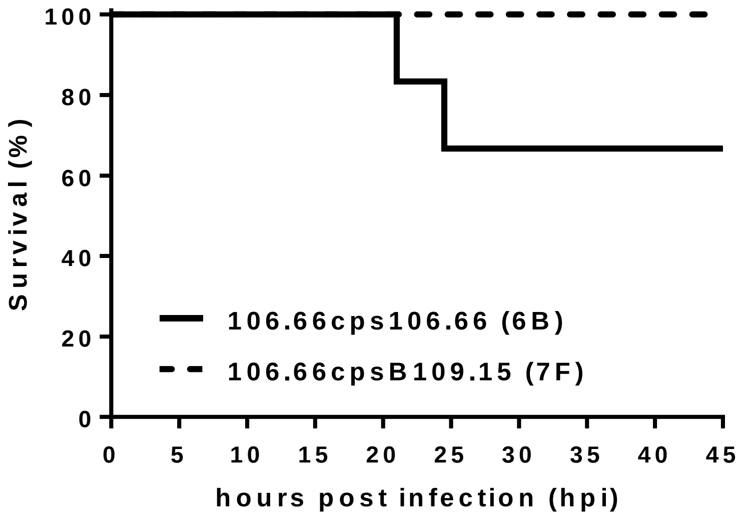

Supplementary Figure S1 – Survival curves during acute bacterial meningitis in animals infected by intracisternal inoculation with a backtransformant of serotype 6B or an alternative capsule switch mutant of serotype 7F. Switching the capsule of wild type strain 106.66 from serotype 6B to 7F (creating capsule switch mutant 106.66cpsB109.15) allowed more survival than in the rats inoculated with the backtransformant 106.66cps106.66 (serotype 6B).

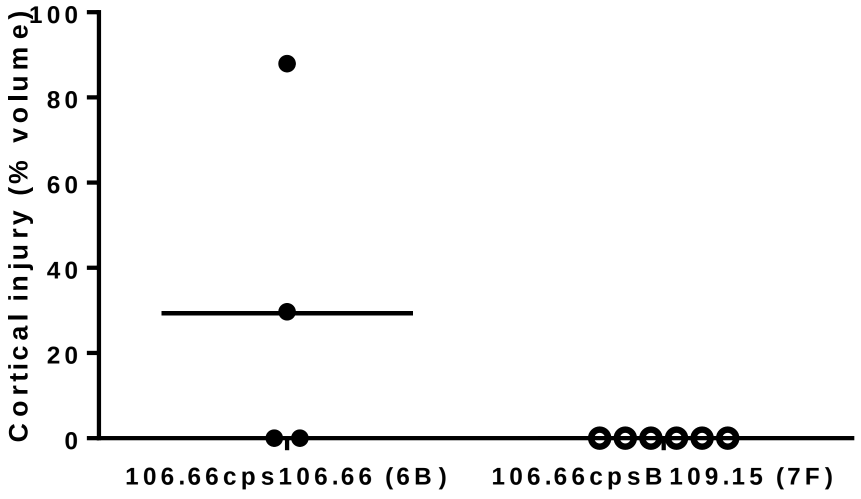

Supplementary Figure S2 - Cortical injury was quantified in rats which survived to 45 hours post infection. The volume of cortical injury was less in rats infected with the capsule switch mutant 106.66cpsB109.15 (serotype 7F) than the backtransformant serotype 6B strain, 106.66cps106.66.

Supplementary Figure S3

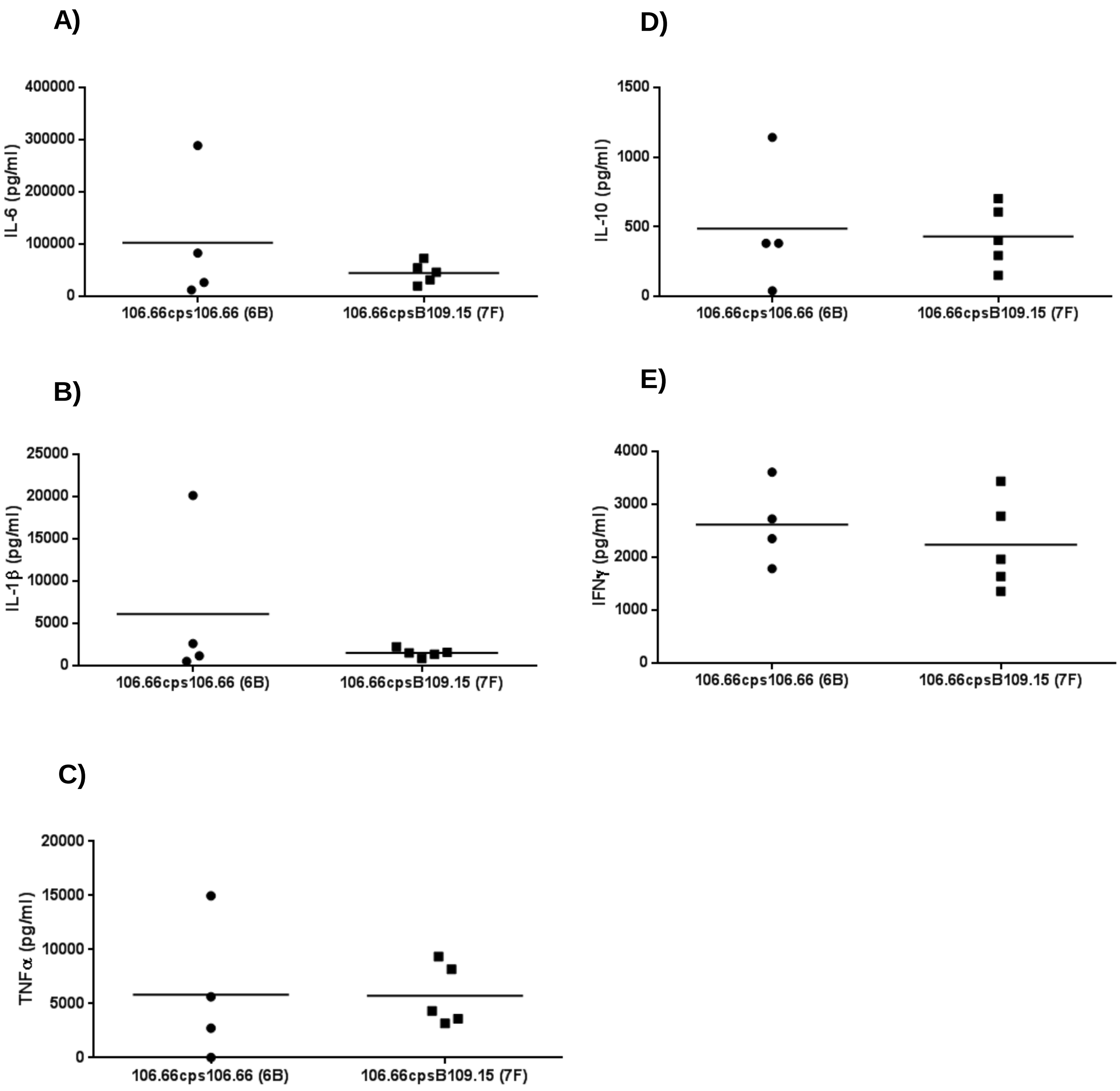

Supplementary Fig S3 - Cytokine concentrations in CSF at 21 post infection.

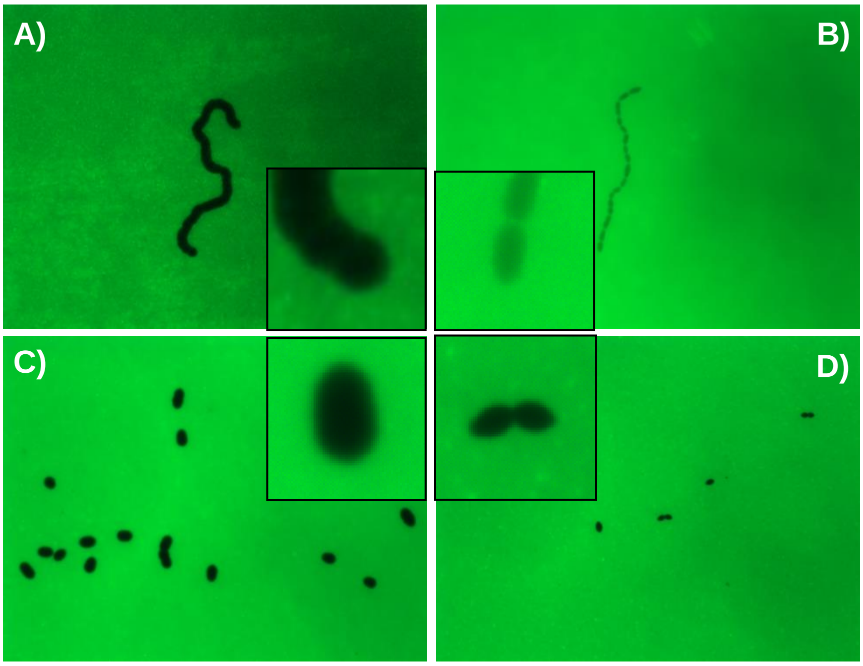

Supplementary Figure S4 – FITC-dextran analysis of capsule thickness. Capsule thickness was determined in the backtransformant 106.66cps106.66 (serotype 6B) (A and C) and capsule switch mutant 106.66cpsB109.15 (serotype 7F) (B and D) growing in BHI before inoculation of rats (A and B) and in CSF recovered from rats 21 hours after infection (C and D) showing that the serotype 6B, but not the 7F, strain can maintain a thick capsule in CSF. Original magnification = 630 X, insets show close ups of outer images. All outer images are to the same scale as each other and all insets are to the same scale as each other.
